# Peer Interaction Does Not Always Improve Children’s Mental State Talk Production in Oral Narratives. A Study in 6- to 10-Year-Old Italian Children

**DOI:** 10.3389/fpsyg.2016.01669

**Published:** 2016-10-25

**Authors:** Giuliana Pinto, Christian Tarchi, Lucia Bigozzi

**Affiliations:** Department of Education and Psychology, University of FlorenceFlorence, Italy

**Keywords:** mental state talk, peer interaction, storytelling, narrative competence, theory of mind

## Abstract

Joint narratives are a mean through which children develop and practice their Theory of Mind (ToM), thus they represent an ideal means to explore children’s use and development of mental state talk. However, creating a learning environment for storytelling based on peer interaction, does not necessarily mean that students will automatically exploit it by engaging in productive collaboration, thus it is important to explore under what conditions peer interaction promotes children’s ToM. This study extends our understanding of social aspects of ToM, focusing on the effect of joint narratives on school-age children’s mental state talk. Fifty-six Italian primary school children participated in the study (19 females and 37 males). Children created a story in two different experimental conditions (individually and with a partner randomly assigned). Each story told by the children, as well as their dialogs were recorded and transcribed. Transcriptions of narratives were coded in terms of text quality and mental state talk, whereas transcriptions of dialogs were coded in terms of quality of interaction. The results from this study confirmed that peer interaction does not always improve children’s mental state talk performances in oral narratives, but certain conditions need to be satisfied. Peer interaction was more effective on mental state talk with lower individual levels and productive interactions, particularly in terms of capacity to regulate the interactions. When children were able to focus on the interaction, as well as the product, they were also exposed to each other’s reasoning behind their viewpoint. This level of intersubjectivity, in turn, allowed them to take more in consideration the contribution of mental states to the narrative.

## Introduction

Research into the development of children’s mental state understanding has recently focused on mental state talk in social interactions as a powerful tool to both explore and foster Theory of Mind (ToM). Mental state talk is defined as that is the set of words used by children to attribute thoughts, feelings, emotions, and desires to people, when referring to either themselves and other people (Bretherton and Beegley, 1982). Mental state talk is facilitated by interactional contexts in which young children communicate with other people about thoughts and feelings. In this study, we will analyze the effect of joint story-telling on children’s mental state talk. Creating a learning environment for storytelling based on peer interaction, does not necessarily mean that students will automatically exploit it by engaging in productive collaboration, thus it is important to explore under what conditions peer interaction promotes children’s ToM. Our understanding of such conditions is limited as most of the studies conducted on joint storytelling have focused on adult-child interactions, rather than on peer interactions. Moreover, prior studies on children’s ToM have mainly focused on its cognitive aspects and on preschoolers. This study extends our understanding of social aspects of ToM, focusing on the effect of joint narratives on school-age children’s mental state talk.

### Theory of Mind and Mental State Talk

Children’s ToM includes several basic skills, that is recognizing emotions, making a distinction between physical and mental entities, appreciating the casual link between perception and knowledge, understanding how desires and beliefs influence behavior, and understanding how beliefs affect behavior (Wellman, 1990; Bulgarelli et al., 2015). The strict interconnection between language and children’s understanding of other people’s mental states has led several scholars to use children’s mental state talk as an indicator of their ToM (Dunn and Hughes, 1998; Astington and Baird, 2005; Symons et al., 2005; Antonietti et al., 2006). Mental state talk includes terms that children use to attribute physiological (e.g., being hungry), perceptual (e.g., see), willing (e.g., desire), emotional (e.g., anger), cognitive (e.g., knowing), moral (e.g., judge), and socio-relational (e.g., helping) state to others (Bretherton and Beegley, 1982; Symons, 2004).

Several studies have used mental state talk as a measure of ToM, for instance to analyze maternal mind-mindedness (Meins et al., 2002), mother–child conversations (Ruffman et al., 2002), conversations between young friends (Hughes and Dunn, 1998) and siblings (Brown et al., 1996), and autistic children (Tager-Flusberg, 1992; Happé, 1994; Capps et al., 2000). A few studies have also validated mental state talk by finding significant correlation scores with standardized measures of ToM, such as the false-belief task (Peterson and Slaughter, 2006; Hughes et al., 2011; Accorti Gamannossi and Pinto, 2014). Thus, evidence from the typically and atypically developing population confirm mental state talk as a reliable indicator of children’s understanding of other people’s ToM.

Mental state talk brings some advantages with respect to more traditional assessments of ToM (e.g., ‘false belief task,’ Wimmer and Perner, 1983): it is a more ecological instrument as it relies on children’s spontaneous production; it allows us to include and analyze several mental states (e.g., desires and feelings, besides the cognitive-related aspects of ToM); it allows us to study the development of ToM in school-age children, since it does not reach a ceiling as other measures do (Wellman and Liu, 2004). Previous studies have demonstrated that individuals’ mind-reading ability grows with age, even beyond school years (adolescence and young adulthood, Valle et al., 2015; and adulthood and elderly age, Cabinio et al., 2015).

### Mental State Talk in Narratives

Narratives represent an ideal context to analyze children’s mental state talk, as through them children develop, practice, and re-describe their ToM (Guajardo and Watson, 2002; Accorti Gamannossi and Pinto, 2014), as is also confirmed by neuro-psychological studies (Marchetti et al., 2015). According to the re-description theory (“representational redescription,” Karmiloff-Smith, 1995), the human mind first develops by learning a process, and then further develops by turning the information that *is* in the mind into explicit knowledge *to* the mind. In this way, processes increase the flexibility of the knowledge we possess. In other words, the mind re-describes its knowledge by representing in different formats what it is internal stored. Re-description theory applies to ToM too. When children are in the process of understanding mental states, they need to understand that a certain event can be represented and viewed differently (Qu et al., 2015). Thus, children’s ToM might be improved by promoting children’s representation, whit the support of narrative tasks.

Children’s development of narrative competence begins early and increases significantly during school years (Makinen et al., 2013). In primary school, children begin to tell or write stories with a basic and conventional macrostructure, which includes initiating events, several interlinked episodes, goal-directed actions, internal responses, and a final resolution (Stein and Glenn, 1982; Gelmini-Hornsby et al., 2011; Squires et al., 2014). Thus, children need advanced mental state talk to create a narrative centered around a protagonist’s intentions and subsequent actions (Pelletier and Beatty, 2015). The relationship between narrative competence and mental state talk develops in particular during primary school years. Generally primary school children tell stories as a list of actions (Carnine et al., 1982; McConaughy et al., 1984), but if they possess a certain level of mental state talk, which allows them to connect action with consciousness, then they are also able to integrate the plot actions with the characters’ mental states (Pelletier and Astington, 2004). Moreover, if the characters’ intentions are explicitly stated, primary school children are able to identify the characters’ mental states (Feathers, 2002). Pelletier and Beatty (2015) examined children’s developing understanding of Aesop’s fables from Kindergarten through Grade 6, and found that as children grow, they are increasingly able to understand fables through their mental state talk, beyond the contribution of general vocabulary. According to Dyer et al. (2000) it is possible that narratives themselves can be an important source of mental state information. The authors analyzed 90 children’s books and found that they included high rates of mental state terms, regardless of the children’s age (they compared books aimed at 3- to 4-year-olds vs. books aimed at 5- to 6-year-olds). They also noted that pictures instead did not represent any mental state, nor did they refer to mental states mentioned in the text.

The development of ToM is particularly facilitated by communication between young children and other people (e.g., mother, father, siblings, peers, and the like) about others’ mental states (Symons, 2004), also through the effect of social shared norms (Massaro et al., 2014). A specific case of interpersonal discourse about mental states is represented by joint narratives. In preschool, kindergarten and school children are exposed to narratives through joint story-telling or story-reading activities. Besides being an activity in which children naturally engage, joint story-telling represents one of the ways in which individual performances can be improved. The effect of peer interaction on children’s mental state talk is explained by several mechanisms. Firstly, peer learning is strictly interrelated with intersubjectivity. The two partners need to achieve a certain degree of intersubjectivity, which can be negotiated or achieved through mutual adjustments (Devescovi and Baumgartner, 1993). Intersubjectivity is strictly interrelated with mental state talk too (Symons, 2004). According to the literature, two conceptual traditions on development psychology focused on intersubjectivity in a meaning co-construction activities: Piaget’s socio-cognitive conflict hypothesis, and Vygotsky’s internalization hypothesis. According to the former perspective, in a joint activity an individual has to take the perspective of the other participant as well, rather than just dealing with his/her own one (Mugny and Doise, 1978). If the two participants are able to achieve a mutual understanding of the activity, then they can achieve a new, and more advanced perspective on the problem. According to Vygotsky internalization process (1978), higher-level processes appear fists at an interpsychological level, and through it are transformed into intrapsychological processes. Children’s participation in interpersonal discourse about the thoughts and feelings of other people facilitates the internalization of the reasoning about mental states, which implies a cognitive reorganization of their own ToM (Symons, 2004). Actually, these two perspectives can be considered as complementary, if we focus on the cooperation between partners, rather than simply the presence of a partner (Kruger, 1993). Both perspectives, although focusing, respectively, on conflict and cooperation, claim that children are able to benefit from a joint activity if they engaged in an extended discourse that explores the reasoning behind the various viewpoints being presented (Kruger, 1993). In this way, the two participants are introduced to each other’s intentions and thoughts on the activity, with a beneficial retroactive effect on their own mental state talk. On the other hand, also the type of task assigned to students has fundamental implications for the efficacy of peer interaction (Slavin, 2004). An exploration of the levels of participation allows us to explore interactional patterns and the source of interaction. In other words, it allows us to understand to what extent students engage in conversations, who initiates the conversations, and whether the response aims at developing the meaning-construction endeavor or rather providing some feedback to the partner. Instead, an exploration of the use of language allows us to analyze the semiotic tools used by participants to mediate the social construction of meaning. Children could engage in a conversation to negotiate meaning, provide and/or justify their perspective, share personal experiences or relevant information, managing the interaction, expressing an agreement/disagreement on what the partner said, evaluating the partner’s contributions to the meaning-making process, and the like. As previously described, narrative represent a perfect outlet for children to reflect on the character’s inner states of mind, providing an ideal context for peer learning to positively influence children’s own mental state talk. In a joint story-telling task, narratives become object of metacognitive reflection: talking about a narrative means talking about ToM.

The understanding of the ways through which children’s mental state talk in primary school can be improved is affected by a few limitations. Firstly, as Hughes et al. (2007) noted few studies have explored school-age children’s mental state talk (Lecce et al., 2010; Longobardi et al., 2016). As with what happened with traditional forms of ToM assessment, most studies on children’s mental state talk have generally explored preschoolers. This is particularly concerning, since several components which have an effect on mental state talk develop during school years (e.g., expansion of vocabulary, working memory, referential communication, and the like). Moreover, schooling introduces a new set of experiences into the child’s life, which create a new set of applications of mental state talk in everyday life (e.g., more social settings).

Secondly, studies in this area have focused especially on parent–child interactions (e.g., Adrian et al., 2005), conversation between siblings and/or friends, but they have rarely explored the facilitation of peer-interaction practices promoted in school, in which students are working together toward a convergent outcome. This is particularly surprising, considering the bulk of research available on the efficacy of peer-assisted learning (Ginsburg-Block et al., 2006; Riese et al., 2012). Such practices are often promoted in school for their positive effects on academic achievements in several different learning processes (Palincsar and Brown, 1984; Ginsburg-Block et al., 2006; Tarchi and Pinto, 2015). In particular, narratives allow us to explore the effect of peer-interaction on an open-ended school activity, which is particularly interesting as it provides children with more opportunities to negotiate meaning and exchange information (Tarchi and Pinto, 2015).

Thirdly, prior studies on socio-cognitive conflict and peer learning (e.g., Mugny and Doise, 1978) have emphasized the importance of taking into consideration children’s levels of individual competence to assess the magnitude of the improvement due to working with a partner. For instance, prior studies found that a socio-cognitive conflict between children is most likely to foster progress in a specific process if children are at the moment of initial elaboration or emergence (Mugny et al., 1981). Most of the studies on children’s mental state talk have assessed it in interactional contexts, but without untangling the relationship between individual and joint mental state talk performance. Moreover, when interacting, each child reciprocally influences each other in their use of mental state talk. However, previous studies demonstrated that children’s mental state talk, generally highly correlated to performances in ToM standardized tests when assessed though an individual task, decrease the strength of this correlation when interacting with older partners (Symons et al., 2005). On the other side, children might use more mental state talk when interacting with peers, rather than with older partners (Dunn, 2000). Thus it is important to explore under what conditions peer interaction promotes children’s ToM. Some studies focused on the individual levels of participants, with two different approaches. According to the peer tutoring approach, peer learning is effective when there is a discrepancy in individual mastery of the target skill (Topping, 2005). Instead, according to the reciprocal peer learning approach, peer learning activities are mostly successful when the two members have similar levels in the target skill and scaffold each other (Duran and Monereo, 2005). In this study, we assessed children’s mental state talk twice, in an individual and in a joint condition.

Lastly, from past studies on peer learning we know that creating a learning environment for storytelling based on peer interaction does not necessarily mean that students will automatically exploit it by engaging in productive collaboration (Prangsma et al., 2007). Prior studies on the discursive practices in peer-interaction educational contexts have put emphasis on both the level of participation in the discourse and the participants’ use of language (Kovalainen and Kumpulainen, 2005; Tarchi and Pinto, 2015).

A few studies have investigated the relationship between the quality of the interaction and mental state talk. Hughes et al. (2006) studied the quality of sibling interactions in relation to children’s mental state talk. One hundred and one families participated in the study, which included 111 2-years-olds and 111 female siblings, for a total of 61 same-sex dyads and 50 opposite-sex dyads. Dyads were video-taped during a 2-h play session at home. Transcripts were coded for presence of mental state talk (referred as inner state talk in the original article). The frequency of mental state talk was significantly correlated with video-based ratings of reciprocal play, also when effects of age, verbal ability and ToM performance were controlled. O’Connor and Hirsch (1999) investigated whether adolescents’ understanding and attribution of mental states was a function of the quality of the relationship, rather than a context-independent characteristic of the individual. Participants were presented with six school situations through a semi-structured interview to assess their mentalising about teachers. Two factors were manipulated to verify the context-dependence hypothesis, most liked compared with least like teacher, and self compared with other student. According to the results, early adolescents exhibit a more advance understanding and attributing of mental states to the behavior of teachers who they like, compared to the ones who they do not like. An indirect measure of the relationships between quality of interaction and mental state talk derives from a study conducted by Meins et al. (2006), who explored 7- to 9-year-old children’s mental state talk in two tasks, book narration versus describing a friend. Children’s mental state talk scores correlated between the two tasks, even after the effects of age and verbal ability were controlled. According to the authors, children’s mental state talk in non-interactional situations generalizes across relational contexts. Furthermore, their mental state talk measures did not correlate with ToM measures, whereas previous studies found that interactional measures of mental state talk were related to ToM. One explanation of this discrepancy could depend on the different ages at which these associations have been explored. Generally, children’s mental state talk in interactional contexts has been studied in preschoolers, thus their developing ToM could have constrained their mental-state reasoning capacities. In older children ToM capacities are more advanced and might no longer influence children’s mental state talk. Alternatively, in preschoolers the association between children’s mental state talk and ToM might be mediated by the mind-mindedness of their partner. This study contributes to the research area on the relationship between the quality of the interaction and mental state talk by exploring mental state talk produced by school-age children interacting with a class-mate in comparison to individual levels of mental state talk.

### Aims of the Study

The aim of this study was to analyze the effect of a peer-interaction condition on mental state talk through a joint narrative task. Consistently with Vygotsky’s internalization hypothesis, participating in a joint narrative task might facilitate children’s development of mental state talk and, in turn, foster a cognitive reorganization of their own ToM (Symons, 2004). Also, consistently with the socio-cognitive conflict hypothesis, peer learning stimulates children to talk about the story, the plot, the characters’ intentions, actions, and internal responses. Talking about a narrative makes the narrative itself an object of a metacognitive reflection.

This study addressed the limitations of the literature by (i) exploring ToM through mental state talk in school-age children, (ii) while engaged in a peer learning task (story-telling in school), (iii) with a focus on the contribution of children’s individual mental state talk, the discrepancy between mental state talk of the two members of a couple, and the quality of the interaction during the joint story-telling task.

Several studies supported the efficacy of peer learning on several aspects of the child’s psychology (Ginsburg-Block et al., 2006; Riese et al., 2012), and emphasized the importance of the interaction with others for the development of ToM (Symons, 2004). Nevertheless, several studies also pointed out that peer interaction does not always produce an improvement in children’s performances, if certain conditions are not satisfied (Devescovi and Baumgartner, 1993; Slavin, 2004; Prangsma et al., 2007). Thus, we investigated whether the efficacy of peer interaction on mental state talk was systematic or not. Specifically, we explored the following conditions of efficacy:

(i) prior studies on socio-cognitive conflict suggested that peer interaction might be effective in fostering progress in a process if children are in an early stage of development (Mugny and Doise, 1978; Mugny et al., 1981), thus we expected peer learning to be effective when children’s individual levels in mental state talk are low;
(ii) prior studies on peer learning have supported the notion that children can progress in a specific skill if they are working with a more competence peer (Topping, 2005), thus we expected peer learning to be effective in the couples with the higher levels of discrepancy between children’s individual levels of mental state talk;
(iii) prior studies on peer learning have widely emphasized the importance for partners to engage in productive discussions with a high level of intersubjectivity (Devescovi and Baumgartner, 1993; Symons, 2004; Kovalainen and Kumpulainen, 2005), thus we expected peer learning to be more effective in couples that were able to engage in interactions characterized by a higher quality of the dialogs.

## Materials and Methods

### Participants

Sixty-four Italian children participated in the study (23 females and 41 males). Eight children were excluded from the study as they did not participate in either the individual story-telling or the joint story-telling task. The final sample included 56 participants. Participants were randomly selected from one predominantly middle-class primary school located on the outskirts of Florence. Four classes were involved (**Table 1**).

**Table 1:** Description of the sample: total number, age, distribution of males and females, and mental state talk performance in individual and joint condition (mean and standard deviation).

Grade	*n*	Age Mean	Males	Females	Individual narrative	Joint narrative
1	12	6.75 ± 0.45	10	2	0.05 ± 0.02	0.11 ± 0.02
2	14	7.71 ± 0.47	6	8		
4	14	9.79 ± 0.43	8	6	0.07 ± 0.02	0.14 ± 0.10
5	16	10.69 ± 0.48	12	4	0.06	0.08
Total	56	8.88 ± 1.64	36	20	0.06 ± 0.02	0.12 ± 0.07

At the time of the study, no participant was diagnosed with a physical or mental disability, nor was included in a diagnostic process, or identified by the teachers as having special educational needs. Parents and school authorities, as well as the children themselves, gave consent to participate in the study. Regarding the Italian educational system, children start formal teaching of literacy at the age of six with entry to primary school and finish it when they conclude the last or fifth grade, at the age of 10 or 11.

### Procedure

Participants were asked to produce oral stories under two different experimental conditions: (a) a free story production by a single child; (b) a free story production by a couple: two children of the same gender constructed and told an invented story together. Joint-narrative partners were randomly assigned. The order of the two tasks was counter-balanced. Each story told by the children, as well as their dialogs were recorded and transcribed. For joint narratives, the dialogs and the story were separated and considered as distinct set of data. The researcher, in agreement with the teachers, at first, explained the story-telling tasks to the entire class so as to reassure the children and promote a climate of trust. Children were asked to make up a story without any book or visual materials or topic to guide them. As a consequence, children generated stories with a very different content. It is important to notice that individual and joint story-telling are daily school activities, since they are often used by teachers, making them an ecologically valid method to explore children’s mental state talk. We included an example of narrative production of a couple of children from 1st grade (two individual narratives and one joint narrative) as Supplementary Table S1. After that, the activity continued in a room adjacent to the classroom both with the individual children and with the couples. First phase, a free story production was requested from the child (Task 1): “I would like you to tell me a story.” Second phase, a free story production was requested from a couple of children (Task 2): “I want you and your partner to tell me a story invented by you together.” In the joint condition, children could plan their performance how they preferred. Some first planned and agreed on the title and/or plot, others just start telling the story and interacted during the construction of the story. Each child, as well as the couples, stayed with the researcher from 15 to 30 min and every story was recorded. Overall, we collected 56 stories and 28 stories told by two children together. The data collection took place in agreement with the school and following the requirements of privacy and informed consent requested by Italian law (Legislative Decree DL-196/2003). Regarding the ethical standards for research, the study referred to the last version of the Declaration of Helsinki (World Medical Association, 2013). The present study was approved by the Ethical Committee of the Department of Psychology at the University of Florence, Italy.

### Coding Systems

Two independent judges coded the narratives in terms of narrative competence and mental state talk in individual and joint narratives, and quality of dialog in joint narratives. Inter-rater agreement scores were all acceptable (*k* > 0.70).

#### Mental State Talk

Mental state talk was analyzed by identifying terms and expressions referring to mental states (adapted from Bretherton and Beegley, 1982). In particular, we identified the following categories: perceptual-physiological states, emotional states, willingness states, cognitive states, and moral and socio-relational states (**Table 2**).

**Table 2:** Description of the coding system for mental state talk (adatpted from Bretherton and Beegley, 1982).

Category	Description	Examples
Perceptual and physiological and states	Terms representing perceptual and physiological states that might influence our behavior (such as hunger and thirst) and describe how we perceive the world	Being hungry, eating, drinking, being born, being ill, watching, listening, smelling, recognizing, feeling bad, felling hot/cold, noticing
Emotional state	Terms describing our feelings and emotions	Happy, pretty, nice, kiss, caressing, cuddle, hug, like, caring, sad, angry, annoyed, ugly, scared, crying, screaming, getting bored, worrying, complaining
Willingness state	Terms describing what we want to achieve and do	Willing, can, hoping, achieving, letting, trying, looking for, ordering
Cognitive state	Terms representing what we cognitively think	Knowing, thinking, understanding, remembering, forgetting, clever, paying attention, true, false
Moral and socio-relational state	Terms representing our moral perspective and the relationships between characters	Good, having to, reprimanding, promising, giving thanks, recommending, obeying, joking, helping, alone, becoming friends, abandoning, tricking

#### Narrative Competence

Children’s narrative competence was assessed in terms of structure, cohesion, and coherence, using a coding scheme developed by Spinillo and Pinto (1994), and adapted by Pinto et al. (2015).

##### Structure

On the base of the presence, absence or/and combination of fundamental elements of a story (title, conventionalized story opening, characters, setting, problem, central event, resolution, and conventionalized story closing), children’s productions were given an index score ranging from 0, “non-story,” to 5, “complete story” (see Supplementary Material for details and examples on the narrative coding system, Supplementary Table S2).

##### Cohesion

Causal and temporal linguistic connectives were counted. Examples of causal connectives are: thus, because, therefore, it follows that, to this aim, as things stand, and the like (e.g., The fox wanted to eat the chicken. **To this aim**, the fox decided to hide). Examples of temporal connectives are: after, before that, at the beginning, suddenly, soon, and the like (e.g., Suddenly, the two boys heard a noise). Based on the number of connectives per total number of words, we assigned the narratives to four categories of cohesion: absent; low (the ratio of connectives/words was below the 33rd percentile); medium (the ratio of connectives/words was between the 33rd and 66th percentiles); and high (the ratio of connectives/words was above the 66th percentile). Absent was assigned a score of 0, low a score of 1, medium a score of 2, and high a score of 3.

##### Coherence

The number of incongruences were identified (sentences introduced by an adversative even though it did not contradict the previous sentence, such as: *the monsters did not want to make peace*, ***but*** *the monsters wanted to attack*). Based on the number of incoherencies per total number of propositions, we assigned the narratives to four categories of coherence: absent; low (the ratio of incoherencies/propositions was below the 33rd percentile); medium (the ratio of incoherencies/propositions was between the 33rd and 66th percentiles); and high (the ratio of incoherencies/propositions was above the 66th percentile). Absent was assigned a score of 0, low a score of 1, medium a score of 2, and high a score of 3.

#### Quality of dialogs

The quality of dialogs was analyzed in terms of discourse moves and communicative functions (Kovalainen and Kumpulainen, 2005).

##### Discourse moves

The analysis of discourse moves shows the participatory roles of each member in collective meaning making. The units of analysis are participants’ utterances. We coded three types of discourse moves: children’s initiation moves, that is utterances used to open a discourse on a particular topic; children’s response moves, that is utterances that elaborated other initiations or responses; and children’s follow-up moves, that is utterances that provided feedback on the ongoing interaction. This analysis allowed us to explore to what extent children engaged in dialogs, rather than producing solo-utterances, and what was the role of the experimenter.

##### Communicative functions

The analysis of communicative functions focalizes on the message unit and permits us to explore the nature of the interaction and its construction in ongoing interactions. The units of analysis are participants’ utterances. We coded nine categories of communicative function (**Table 3**).

**Table 3:** Analysis of communicative functions.

Function	Description	Example
Evidence negotiation	Asking for and presenting evidence, justification or reasons	*“Yes, short like that, a couple of lines are enough for me”* (Male, first grade).
Defining	Asking for and providing definitions, elaboration, clarification or demonstration	*“Ok, first we should agree on the title”* (Male, fourth grade).
Experiential	Asking for and sharing personal experiences, feelings or examples from one’s own life	*“You know, this has really happened to me”* (Female, second grade).
View sharing	Asking for and expressing views, opinions or perspectives	*“I think a good story should end with ‘happily ever after”’* (Male, first grade).
Information exchange	Asking for and providing information, solutions or observations	*“Isn’t this the story that the teacher told us in class the other day?”* (Female, second grade).
Orchestration of classroom interaction	Taking charge of the interactive management of speaking turns	*“Come on, go on with the story please”* (Male, fourth grade).
Confirming	Acknowledgment and acceptance of the topic of interaction	*“Shall we create a story on animals?” “Yes, it is a good idea. Once upon a time …”* (Females, second grade).
Evaluation	Assessment of the contributions to meaning-making	*“Come on, put some effort, you are driving us away from the story”* (male, fifth grade).

### Data Analysis

Mental state talk was divided by the fluency of the participants’ productions: the total number of words used to tell the stories was counted to create ratios, standardize participants’ performances, and check for the potentially confounding effect of narrative length. Ratios were also calculated for cohesion and coherence score, dividing raw scores by the total number of words. Following, mental state talk scores were transformed into percentiles. There are several ways to explore children’s narrative competence, adopting both continuous data (Haden et al., 1997; Fivush et al., 2006), and categorical data (Bigozzi and Vettori, 2015; Pinto et al., 2015, 2016b). In this study, narrative competence variables (i.e., mental state talk, structure, cohesion, and coherence) were re-coded into a 3-point scale using the percentile distribution: the first point was for scores lower than the 33rd percentile, the second point for scores between the 33rd and the 66th percentile and, finally, the third point corresponded to scores higher than the 66th percentile. Each variable was re-coded coherently with this positional criteria, both for individual and for joint narrative tasks.

To verify whether the joint condition systematically improved students’ mental state talk when compared to their individual performances we identified incremental and decremental subjects. To this aim, we compared the individual and joint performances of each subject, and identified two groups: individuals who incremented their mental state talk from the individual to the joint condition (incremental), and individuals that decremented their mental state talk from the individual to the joint condition (decremental) (**Table 4**).

**Table 4:** Frequencies of decremental and incremental individuals/couples (total scores and divided by grade).

Grade	Individuals			Couples		
	Decremental	Incremental	Total	Decremental	Incremental	Total
1st	2	8	10	1	3	4
2nd	8	6	14	2	0	2
4th	6	8	14	2	4	6
5th	12	4	16	5	1	6
Total	28	26	54	10	8	18

Since prior research showed that children’s narrative competence develops throughout primary school (Bamberg, 1997), we verified the influence of children’s narrative competence on the efficacy of peer interaction on mental state talk. To this aim, we compared performances in structure, cohesion, and coherence of incremental children versus decremental children. Then, we verified whether the joint condition is particularly effective for individuals for low levels of mental state talk. We tested the frequency of participants’ distribution in the three groups through a binomial statistical test.

To verify the conditions under which joint narratives have a beneficial effect on children’s mental state talk, we changed the unit of analysis from the individual to the couple, and proceeded to identify incremental and decremental couples. A couple was defined as incremental if the percentile score in the joint condition was higher than the scores obtained by the two participants of the couple in the individual condition. A couple was defined as decremental, if the percentile score in the joint condition was lower the scores obtained by the two participants of the couple in the individual condition (**Table 4**). We explored two conditions through a series of Mann–Whitney *U* tests: (i) whether the joint condition is particularly effective for couples made up of individuals with discrepant individual performances in mental state talk; and (ii) whether incremental couples were engaged in interactions of higher quality than decremental couples were. For all statistical analysis, the effect-size was estimated (Fritz et al., 2012).

## Results

Descriptive statistics for mental state talk and narrative competence in the individual and joint condition are reported in **Table 5**. Descriptive statistics for quality of interaction in the joint condition are reported in **Table 6**

**Table 5:** Descriptive statistics for mental state talk and narrative competence (ratios: mental state term/number of words): Mean (M), standard deviation (SD), median (Mdn), skewness (Skw), and kurtosis (Kur).

Variables	Individual condition					Joint condition				
	*M*	*SD*	Mdn	Skw	Kur	*M*	*SD*	Mdn	Skw	Kur
**Mental state talk**										
Perceptual	0.026	0.022	0.022	0.618	–0.377	0.030	0.023	0.026	0.711	–0.344
Emotional	0.006	0.010	0.008	1.763	4.529	0.009	0.018	0.003	4.123	19.231
Willingness	0.014	0.015	0.009	1.66	2.573	0.015	0.015	0.011	1.559	2.833
Cognitive	0.017	0.025	0.010	2.99	11.257	0.007	0.012	0.002	3.465	14.882
Socio-relational	0.012	0.014	0.009	1.943	4.335	0.013	0.015	0.009	2.201	5.099
Total	0.075	0.038	0.076	–0.030	0.234	0.074	0.023	0.069	0.828	2.499
**Narrative competence**										
Structure	2.67	1.25	2.75	–0.053	–1.513	2.68	1.12	2.00	0.023	–1.491
Cohesion	0.06	0.02	0.06	0.048	0.256	0.06	0.04	0.06	3.037	13.949
Coherence	0.03	0.05	0	1.583	2.072	0.06	0.05	0.05	0.762	–0.266

**Table 6:** Descriptive statistics for quality of interaction (count of discourse moves and communicative functions): Mean, standard deviation, median, skewness, and kurtosis.

Variables	*M*	*SD*	Mdn	Skw	Kur
**Discourse moves**					
Student’s initiation	1.68	1.56	1.00	1.579	2.615
Student’s response	6.46	4.61	5.00	0.655	–0.644
Student’s feedback	3.14	3.12	2.00	0.941	–0.010
Total student’s moves	11.28	6.98	10.00	0.960	0.666
**Communicative functions**					
Confirm (accept an argument)	1.21	1.83	0.50	1.991	3.798
Give/ask for a definition	4.07	2.83	4.00	0.949	0.350
Assessment of contributions	0.07	0.26	0	3.520	11.183
Negotiation of evidence	0.68	0.90	0	1.359	1.291
Share experience	0.75	1.14	0	1.494	1.401
Give/ask for questions	3.21	3.63	3.00	1.912	4.696
Orchestrate the interaction	0.61	1.29	0	3.173	11.591
Give/ask for opinion	0.04	0.19	0	5.292	28.000

In the individual condition, mental state talk did not correlate with any narrative competence score, namely structure (*r* = 0.14, *p* = 0.31), cohesion (*r* = -0.13, *p* = 0.34), or coherence (*r* = -0.02, *p* = 0.89). In the joint condition, mental state talk correlated with cohesion (*r* = 0.41, *p* = 0.04), but not with structure (*r* = 0.12, *p* = 0.56) or coherence (*r* = -0.06, *p* = 0.77). According to the Mann–Whitney test, the performances in structure (*U* = 359.50, *z* = -0.81, *p* = 0.94, η^2^ = 0.00), cohesion (*U* = 300.00, *z* = -1.11, *p* = 0.27, η^2^ = 0.07), and coherence (*U* = 267.50, *z* = -1.80, *p* = 0.07, η^2^ = 0.18) of incremental and decremental children were statistically similar.

### Effects of Joint Narratives

**FIGURE 1:**
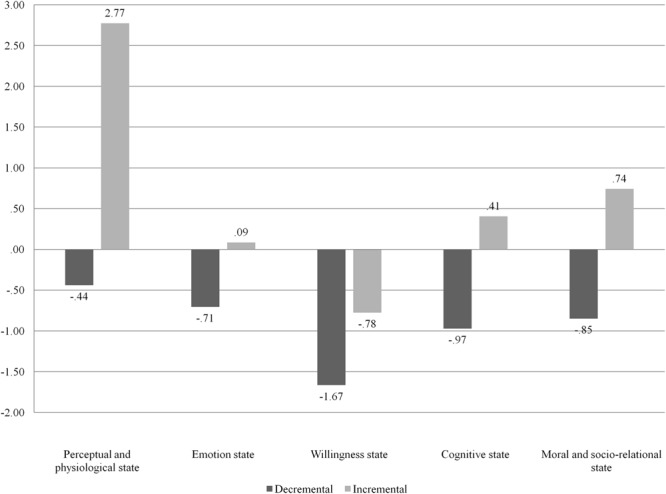
**Patterns of changes of mental state talk categories from the individual to the joint condition**.

The joint condition was not systematically beneficial for all students’ mental state talk performances. The probability of using more mental state talk in the joint condition than in the individual one was not above chance (Binomial test, *p* = 0.89). On a descriptive level, we compared the differences from the individual to the joint performances of incremental versus decremental participants (**Figure 1**). In the joint condition, incremental children are able to increase their use of perceptual, moral, and willingness terms, whereas emotional terms are substantially stable in the two conditions. Instead, incremental children also decrease their use of cognitive terms in the joint condition. Decremental individuals decrease the use of mental state talk in all categories from the individual to the joint condition, with cognitive terms displaying the higher percentage of change.

### Conditions of Efficacy of the Joint Condition

To explore the conditions under which joint narratives increase children’s mental state talk, we changed our unit of analysis to couples (incremental and decremental). To illustrate the differences in incrementation and presence of mental state in the individual narrative across grades, in **Table 1** we report the means of the incremental couples’ mental state talk ratios in the individual and joint condition, for the total sample as well as for each grade.

When analyses were conducted at the individual level, one statistical significant result emerged. According to the Mann–Whitney *U* test, incremental individuals (Rank mean = 20.54) had lower levels of mental state talk in the individual condition than decremental individuals had (Rank mean = 33.96), *U* = 183.00, *z* = -3.14, *P* < 0.01, η^2^ = 0.55.

When analyses were conducted at the couple level, two statistical significant result emerged, both related to differences in quality of interaction. When we compared types of couples on the basis of discrepancy among individual performances in mental state talk of the two members of each couple, the Mann–Whitney *U* test did not report a statistically significant difference (**Table 7**). When we compared types of couples on the basis of quality of interaction (discourse moves, and communicative functions), the Mann–Whitney *U* test showed that incremental couples are characterized by more dialogs initiated by the teacher, and more utterances aimed at orchestrating the interaction than decremental couples are. Although not a significant result, the Mann–Whitney showed a tendency for students in incremental couples to speak more than students in decremental couples (**Table 7**).

**Table 7:** Mean rank comparison between the two types of couples (incremental vs. decremental) in terms of mean discrepancy between individual performances of the two members of each couple and quality of interaction (discourse moves and communicative functions): sample sizes, mean ranks, Mann–Whitney *U* test (*Z*_U_), *p*-value and effect-size (η^2^).

	*U*	*Z*	*p*	η^2^	Mean rank	
					Decremental	Incremental
Discrepancy	23.00	–1.51	0.13	0.13	7.80	11.63
**Discourse moves**						
T’s initiation	17.50	-2.08	0.04	0.24	7.25	12.31
T’s response	26.50	–1.26	0.21	0.09	8.15	11.19
T’s feedback	28.50	–1.03	0.30	0.06	8.35	10.94
T’s total moves	23.00	–1.52	0.13	0.13	7.80	11.63
S’s initiation	23.00	–1.59	0.11	0.14	7.80	11.63
S’s response	25.50	–1.30	0.19	0.09	8.05	11.31
S’s feedback	27.00	–1.18	0.24	0.08	8.20	11.13
S’s total moves	21.00	–1.70	0.09	0.16	7.60	11.88
**Communicative functions**						
Confirming	31.50	–0.87	0.39	0.04	10.35	8.44
Defining	38.50	–0.14	0.89	0.01	9.35	9.69
Evaluation	40.00	0.00	1.00	0.01	9.50	9.50
Evidence negotiations	34.50	–0.53	0.60	0.02	10.05	8.81
Experiential	34.50	–0.58	0.56	0.02	10.05	8.81
Information exchange	35.00	–0.45	0.65	0.01	9.00	10.13
Orchestration of the interaction	17.00	-2.18	0.03	0.26	6.89	11.38
View sharing	35.00	–1.12	0.26	0.07	9.00	10.13

*T, teacher; S, student.*

## Discussion

The aim of this study was to analyze whether a joint narrative condition influenced children’s production on mental state talk. Mental state talk is a valid and reliable indicator of children’s ToM (Dunn and Hughes, 1998; Astington and Baird, 2005; Symons et al., 2005; Antonietti et al., 2006), thus the results of this study can contribute to our understanding of the influence of interactional contexts and discursive practices in school on children’s understanding of other people’s thoughts, beliefs, feelings, and intentions. School peer-interaction practices have a demonstrated positive effect on several aspects of the child’s psychology (e.g., academic performances, O’Donnell and King, 1999; cognitive development, Riese et al., 2012; and social skills, Ginsburg-Block et al., 2006), and we extended this effect to mental state talk. We were interested in the conditions under which a peer-interaction context improves children’s mental state talk.

Firstly, we controlled the effect of narrative competence. Narratives themselves are an important source of mental state talk (Dyer et al., 2000), thus children’s production of mental states could be influenced by their capacity to represent the protagonist’s intentions and subsequent actions (Pelletier and Beatty, 2015). Our resulted indicated that children’s production of mental state talk was unrelated to their competence in producing a narrative with a conventional structure, either in the individual or joint condition. Mental state talk appears to be an independent component of children’s mind, which can be facilitated or hindered by contextual variables, such as a narrative task, but does not overlap with other skills involved by the task itself, such as narrative competence. In other words, children’s mental state talk is activated by narratives, rather than being a by-product of narrative competence. Prior research showed that children’s narrative competence develops throughout primary school (Bamberg, 1997). In this study, we controlled for this potentially confounding effect by comparing incremental and decremental children’s performances in structure, cohesion and coherence. No significant difference emerged, suggesting that children’s developing narrative competence did not play a significant role in supporting mental state talk. Narrative competence and ToM appear to be independent constructs.

The results of this study confirmed that peer interaction does not automatically lead to increased performances, as not necessarily are two students able to engage in a productive collaboration (Prangsma et al., 2007). Before turning our attention to the conditions under which peer interaction produces an increase in mental state talk, let us discuss changes in the patterns of mental state talk from the individual to the joint condition in incremental and decremental couples. In the joint condition, incremental couples increase their use of perceptual and physiological terms, willingness terms, and moral terms. In particular, incremental and decremental couples display the largest difference in the use of moral terms. Thus, peer interaction seems to act on the core component of a narrative. According to Linde (2010), it is the inclusion of a moral meaning that distinguishes a story from a list of events or a chronicle. Interestingly, moral components cannot be completely defined structurally, as confirmed by the lack of correlation between mental state talk and narrative competence, including the structural component. Linde (2010) also added that a narrative can be considered successful if there is an agreement on the moral meaning of a story. Generally, such an agreement should take place between the narrator and the interlocutor, whereas a joint narrative activity requires this agreement to be reached by the two narrators. In this sense, peer interaction might be a reflective tool on the moral aspects of a story and on its dialogical nature.

The other two main differences between incremental and decremental couples in terms of change across the two conditions concern perceptual-physiological terms and willingness terms. As suggested by previous studies Pelletier and Beatty (2015) children need high levels of mental state talk to create a narrative based on the characters intentions and the subsequent actions. Thus, peer interaction might stimulate children to share and negotiate the intentions of the characters of the joint narrative (i.e., willingness states) and the actions connected to such intentions (i.e., perceptual and physiological states). Feathers (2002) stated that if the characters’ intentions are explicitly described in a narrative, then children are abler to identify each mental state present in the story, and peer interaction might contribute to this link.

Once confirmed that peer interaction does not automatically lead to higher performances in mental state talk, we proceeded to explore the conditions under which children increased their mental state talk from the individual to the joint condition. A first variable controlled in this study was children’s individual levels of mental state talk. Prior studies have demonstrated in certain cases, children’s ToM, as assessed by a standardized test, is more strictly related to their individual mental state talk, rather than to the mental state talk produced while interacting with a partner (i.e., older partner, Symons et al., 2005). According to our data, children included in the incremental couples had lower levels of mental state talk in individual narratives than children included in the decremental couples did. Thus the facilitating effect of a peer-interaction condition is confirmed for children who are at the moment of initial elaboration or emergence of mental state talk, in line with prior studies demonstrating the conditions under which group performance is superior to individual performance (Mugny et al., 1981).

A second variable explored in this study to explore the conditions under which peer interaction positively influences children’s mental state talk was discrepancy between the individual mental state talk of the two members of a couple. According to our data, the individual levels of mental state talk of members of incremental couples were not more or less discrepant than the ones of decremental couples. This finding emphasizes that for peer learning to be effective, there is no need to create a couple with an asymmetrical relationship (“peer tutoring;” Duran and Monereo, 2005), a model advocated by Vygotsky, who claimed that problem-solving in interaction with more expert peers allows the child to enter new areas of potential (i.e., zone of proximal development), with both members of the couple benefitting from the interaction by internalizing all the processes enacted during the meaning-constructing discourse (Vygotsky, 1978).

Finally, we examined the interaction between partners in joint narratives in terms of source of interaction and communicative use of language. According to our data, incremental couples interacted more than decremental couples did, as shown by a higher number of interventions by the children. Also, children produced more utterances to orchestrate and regulate the dialog, which is probably the reason why children in the incremental couples interacted more and, in turn, benefitted more from the joint narrative condition. Peer-assisted learning contexts require high levels of intersubjectivity, which needs to be accomplished by mutual adjustments of the two partners (Ginsburg-Block et al., 2006; Tarchi and Pinto, 2015). None of the other comparisons was statistically significant. Students in incremental and decremental couples seemed to interact in a similar way: they mainly interacted to define and elaborate the topic of their narrative, exchanged information and confirmed that they agreed on their partner’s story-lines.

## Conclusion

This study describes the effect of peer-interaction on mental state talk. Our results suggest that a peer interaction intervention is mostly beneficial for children with lower levels of individual mental state talk. This is consistent with a traditional line of research on socio-cognitive conflict emphasizing how children progress as a function of interacting with others is significant when they are in the initial stages of the elaboration of the target process (Mugny et al., 1981). Moreover, interaction played an essential part in the effect of peer-learning. Children who improved their mental state talk in the joint condition have been able to create a high level of intersubjectivity with their partner, as demonstrated by the higher number of interventions to orchestrate the dialog. When focusing on the interaction, as well as the product, children were also able to achieve a mutual understanding of the activity by being exposed to each other’s reasoning behind their own viewpoint (Kruger, 1993). This mechanism appeared to be more important than having students working with a more expert peer (peer tutoring, Duran and Monereo, 2005).

This finding provides useful information for educators: children’s ToM can be improved through children’s engagement in a peer-assisted learning task. Moreover, in agreement with Pelletier and Beatty (2015), we believe that this study also contributes to improving children’s appreciation of narratives, which could be hindered by an impaired understanding of the story characters’ mental states. Furthermore, our results emphasize the importance of the role played by the teacher. Incremental couples were characterized by more interventions by the adult, which scaffolded children’s interactions and co-construction of the story.

This study was affected by a few limitations. Firstly, our results are limited by the small sample size, which determines problems of statistical powers and risks of not finding existing associations between variables. Moreover, the size of our sample sizes did not allow to test the moderation effect of age on the association between peer interaction and mental state talk. Secondly, although several studies used and validated mental state talk as an implicit measure of ToM (e.g., Dunn and Hughes, 1998; Astington and Baird, 2005; Symons et al., 2005; Antonietti et al., 2006), results from this study would be sounder if an explicit evaluation of ToM with a specific test was included. Thirdly, past studies have shown that children’s ToM and mental state talk correlate with other variables, such as executive functions (Bianco et al., 2015). Future studies should include these variables and examine whether the results obtained in this study partially depend on their influence. Fourthly, generalization of results is limited by the research design of this study, in particular by the use of oral narratives. Prior studies have demonstrated the presence of a discontinuity in children’s narrative competence, when writing is introduced (Pinto et al., 2015, 2016b). In primary school children are asked to write their narratives, rather than tell them, but we believe in the importance of keeping oral narratives in primary school too, given their fundamental role in eliciting and organizing children’s ToM through the use of mental state talk (Guajardo and Watson, 2002; Pinto et al., 2016a). Finally, in this study children were allowed to create stories without specific directions. As a consequence, children’s narratives resulted in a wide variety of contents. Prior studies emphasized the influence of the context and instructions on children’s narrative production (e.g., Berman, 1995; Cameron and Hutchison, 2009), and future studies should verify whether also mental state talk depends on the instructions given and the content of the stories produced.

## Author Contributions

All authors listed, have made substantial, direct and intellectual contribution to the work, and approved it for publication.

## Conflict of Interest Statement

The authors declare that the research was conducted in the absence of any commercial or financial relationships that could be construed as a potential conflict of interest.
