# Supplementary material for: Peer Interaction Does Not Always Improve Children’s Mental State Talk Production in Oral Narratives. A Study in 6- to 10-Year-Old Italian Children
Source: Front Psychol. 2016 Oct 25;7:1669. doi: 10.3389/fpsyg.2016.01669 (PMC5078763; doi:10.3389/fpsyg.2016.01669)
Supplement: Supplementary file 1 [file Table_1.DOCX]

Supplementary Material

Peer interaction does not always improve children's mental state talk production in oral narratives. A study in six- to ten-year-old Italian children.

Giuliana Pinto, Christian Tarchi*, Lucia Bigozzi

*** Correspondence:** Corresponding Author: christian.tarchi@unifi.it

# Supplementary Table

## Example of individual and joint narratives

| **Individual narratives** | **Joint narratives** |
| --- | --- |
| Participant 1: The blackboard  Once upon a time, there was a blackboard. When the teacher was writing on it, the blackboard was always saying “no!”. One day, as the teacher was going to write on it, the blackboard run away, and went to another school and in another room. When the teacher came back, he/she did not see the blackboard any longer, and all the children told her that without a blackboard they had to go on holiday earlier. But then the blackboard came back, the teacher laughed and wrote some stuff on the blackboard. The children read what was written on the blackboard and when the teacher wanted them write a full stop, they instead wrote a comma, because the blackboard was telling them not to write a full stop.  Participant 2: The fruit-eater mouse  Once upon a time there was a mouse that was always eating fruit. It was fat, beautiful, and had a little house. Every day it ate fruit, every week. Then it always went into its little house, then it went to the market to buy some fruit, then it went in its little house. The mouse met a little ant and said: “Do you want to live with me?” The ant said “Yes” and then they bought all the fruit that existed in the world. | Participant 1 and 2: The big-eating mouse  The big-eating mouse wants always to eat. There was a mouse that was eating every day, it was fat, round, a mouse that was always eating cheese. Every day as soon as it got up, the mouse ate cheese. It even emptied the basement. And there was a cat that wanted to eat the mouse. Close to its room, that was little, the cat was putting its claws inside to get the mouse. Then the dog came and ate the cat. And the dog became friend with the mouse. And the lion could not find its way to eat the cat. Then the lion found a wolf and ate the wolf. And then the hippopotamus arrived and ate the lion. |
