# Supplementary material for: Peer Interaction Does Not Always Improve Children’s Mental State Talk Production in Oral Narratives. A Study in 6- to 10-Year-Old Italian Children
Source: Front Psychol. 2016 Oct 25;7:1669. doi: 10.3389/fpsyg.2016.01669 (PMC5078763; doi:10.3389/fpsyg.2016.01669)
Supplement: Supplementary file 2 [file Table_2.DOCX]

Supplementary Material

Peer interaction does not always improve children's mental state talk production in oral narratives. A study in six- to ten-year-old Italian children.

Giuliana Pinto, Christian Tarchi*, Lucia Bigozzi

*** Correspondence:** Corresponding Author: christian.tarchi@unifi.it

# Supplementary Table

## Narrative structure coding scheme.

| **Level** | **Type of narrative** | **Structural elements included** | **Example** |
| --- | --- | --- | --- |
| 1 | No narrative | simple description or list of events, objects, or facts | Once upon a time there was a mouse that was always eating fruit. It was fat, beautiful, and had a little house, and was always eating fruit. It was going to the market, buying fruit, and then eating it (*Male, 1^st^ grade*) |
| 2 | sketch narrative | opening, setting, character(s), conclusion or opening, sketch of the problem, and resolution | Once upon a time there was a child who lived in a cave, and wanted to find a treasure. He looked for it, all day long, and eventually he found it, but it was very small, while he thought that there was going to be a lot. So a pirate appeared and said “give me this gold, or I’ll feed you to the sharks!” The child gave it to him, but a monster destroyed the ship and the story ended happily ever after (*Male, 2^nd^ grade*). |
| 3 | incomplete narrative | opening, character(s), problem, and resolution | Once upon a time there was a child, who during summertime every Wednesday was going with another child to seat next to the Watermelon seller to steal two watermelons and run away. This happened for one, two weeks. On the third week, this child got caught on a tree while he was stealing a watermelon, and the other child was at home. The child caught on a tree was taken home by the Watermelon seller, who said that he was stealing the watermelons, and the father and the mother decided that he could no longer go out on Wednesdays, and decided to send him to boarding school (*Male, 5^th^ grade*). |
| 4 | essential narrative | opening, character(s), problem, central event, and resolution | Once upon a time there was a chicken, that was eating corn and a fox was spying on it while it was eating, so on its toes the fox arrived and said: “Hi, what are you doing, chicken?”, and the chicken: “I am … am … eating, what about you? And the fox: “I was looking at you eating” and the fox says goodbye. On another day the fox comes back and tried to arrive unnoticed because it wanted to it the chicken. So it walked without any noise and eats the chicken. Then the fox goes back to its den and sleeps. Then a hunter arrives and sees the fox with a big big belly. The hunter knew that the fox was after chickens, so the hunter cuts the fox open and the chicken can go out and run on the grass, where it could eat happily ever after (*Female, 1^st^ grade*). |
| 5 | complete narrative | title, opening, character(s), setting, problem, central event, resolution, and narrative closing | Once upon a time there was a little vampire, she went out and sw a cave, leading to a secret passage. Here there were some steps, she went up and saw a huge door, she opened it and saw a beautiful garden. Everything was made out of chewing-gum, lollies and ice-cream, it was beautiful. A boy arrived and asked her what her name was. And she said that her name was “Vampiretta.” Suddenly, many children arrived with a big ogre, and the ogre asked to the boy: “what are you doing, son, with this girl? We will keep her as a prisoner.” But the boy was good and he did not want her to be prisoner of the children, so Vampiretta began to run away and found the secret passage and hid. The boy found her in a little corner and they prepared together a plan to defeat the ogre. And they defeated him, because they found out that cats’ meow could defeat him, this was his weakness. They got a cat and put him under his bed at night. He died in a burst of rage, and all the children were freed (*Female, 4^th^ grade*). |
